# Parasites FeS Up: Iron-Sulfur Cluster Biogenesis in Eukaryotic Pathogens

**DOI:** 10.1371/journal.ppat.1003227

**Published:** 2013-04-04

**Authors:** Teegan A. Dellibovi-Ragheb, Jolyn E. Gisselberg, Sean T. Prigge

**Affiliations:** W. Harry Feinstone Department of Molecular Microbiology and Immunology, Johns Hopkins Bloomberg School of Public Health, Baltimore, Maryland, United States of America; University of Wisconsin Medical School, United States of America

## Function and Biogenesis of FeS Clusters

Iron-sulfur (FeS) clusters are among the most ancient and versatile protein cofactors. They are used by a large and diverse group of proteins, serving both structural and catalytic roles. They function in central metabolic processes such as electron transfer, redox chemistry, enzyme catalysis, and sensing environmental or intracellular conditions to regulate gene expression [1], [2]. Ferrous iron and sulfur were readily available in the reducing atmosphere in which life first evolved, but when oxygen levels rose with the advent of photosynthetic algae, these building blocks became scarce. Furthermore, the reactive oxygen species generated as byproducts of aerobic respiration are highly damaging to FeS clusters, and free iron and sulfide released by FeS clusters are, in turn, toxic to cells. For these reasons, complex mechanisms evolved to coordinate and regulate the biogenesis of these simple cofactors, and these pathways are compartmentalized in the endosymbiotic organelles of eukaryotic cells.

All FeS cluster biogenesis pathways follow the same basic principles (reviewed in [2]). The first step is the liberation of sulfur by a cysteine desulfurase, which forms a persulfide intermediate on a conserved cysteine residue. Iron is donated by iron-binding proteins, such as frataxin. The cluster is then assembled on scaffold proteins with the help of electron donors, which are needed for the reduction of sulfur to sulfide. The fully formed cluster is transferred to apoproteins via chaperones that facilitate the correct substrate specificity and proper assembly of the cluster to form the mature holoprotein.

**Figure 1:**
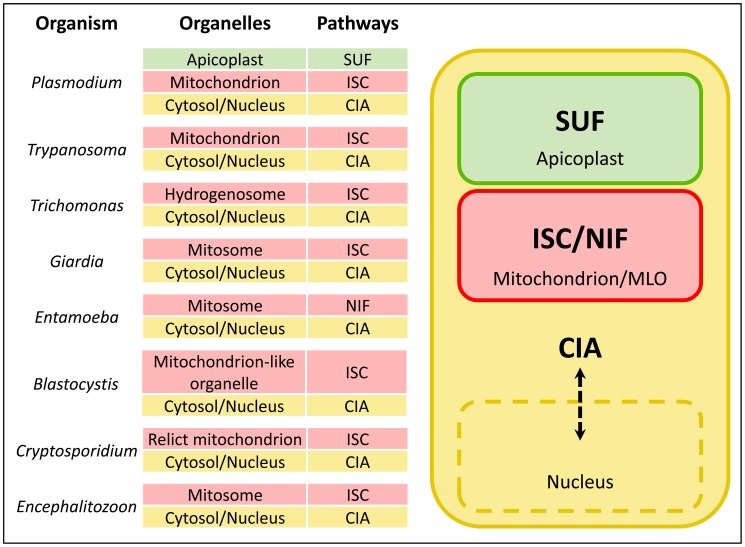
FeS cluster biogenesis pathways in eukaryotic pathogens. Eukaryotic pathogens contain an ISC (Iron-Sulfur Cluster formation) pathway in the mitochondrion or mitochondrion-like organelle (MLO), a CIA (Cytosolic Iron-sulfur protein Assembly) pathway in the cytosol, and plastid-containing organisms such as *Plasmodium* have the SUF (SUlFur mobilization) pathway in this organelle. FeS cluster biogenesis is absolutely essential in mitochondria and MLOs, and is likely the driving force for retention of these organelles. *Entamoeba* is the exception that proves the rule: it has dispensed with the ISC machinery, but replaced it with a bacterial NIF (NItrogen Fixation) pathway acquired by lateral gene transfer that appears to fulfill the requirement for FeS cluster synthesis in the mitosome.

FeS cluster biogenesis pathways are extremely well conserved, and are invariably essential for viability. Among eukaryotic pathogens, all endosymbiotic organelles studied to date appear to contain FeS cluster biogenesis machinery, and, in some cases, this seems to be the sole reason for retention of the organelle. Typically, the ISC (Iron-Sulfur Cluster formation) pathway resides in the mitochondrion, the CIA (Cytosolic Iron-sulfur protein Assembly) pathway functions in the cytosol and nucleus, and plastid-containing organisms have an additional pathway, the SUF (SUlFur mobilization) system (Figure 1). In this article, we will compare the subcellular organization of FeS cluster biogenesis pathways in the diverse organelles of eukaryotic pathogens.

## The SUF Pathway in the Apicoplast Organelle of Apicomplexan Parasites

The unusual apicoplast organelle present in most intracellular protists of the phylum Apicomplexa (which includes parasites such as *Plasmodium*, *Toxoplasma*, *Neospora*, *Babesia*, and *Theileria*) contains an FeS biogenesis pathway as well as a number of FeS-dependent proteins. The apicoplast was acquired through a secondary endosymbiosis event in which a plastid-containing algal cell was engulfed by a eukaryote. This four-membrane-bound organelle has retained a small circular genome, and the metabolic pathways it contains reflect its prokaryotic origin. The existence of the SUF pathway in the apicoplast was predicted by bioinformatics studies [3], [4], and was recently demonstrated experimentally in the malaria parasite *Plasmodium falciparum* [5]. Interestingly, one of the enzymes of the SUF pathway, SufB, is one of only a few nonhousekeeping genes encoded on the apicoplast genome. The SUF pathway is particularly adapted to synthesize FeS clusters under conditions of oxidative stress or iron starvation [6], [7], which was an advantage in the oxygen-rich environment of the photosynthetic algae from which the pathway was inherited. The SUF pathway is thought to provide FeS clusters exclusively for apicoplast-resident proteins, such as ferredoxin, IspG and IspH (both components of the isoprenoid biosynthesis pathway), LipA (lipoic acid synthase), and MiaB (tRNA methylthiotransferase) [8]. Recently, it was demonstrated that the product of the isoprenoid biosynthesis pathway, isopentenyl pyrophosphate (IPP), could sustain growth of malaria parasites when the apicoplast was ablated by antibiotic treatment, suggesting that export of this metabolite to the cytosol is the only essential function of the apicoplast in the blood stages of the parasite lifecycle [9]. Since the SUF pathway supplies FeS clusters to two enzymes of the pathway that produces IPP, this finding also strongly suggests that FeS cluster biogenesis in the apicoplast is essential for parasite viability. Thus, the enzymes of the SUF pathway are attractive candidates for development of new drug targets because of their essentiality as well as their prokaryotic origin, which makes them significantly different from any enzymes found in the host cell.

## The ISC Pathway in Mitochondriate and Amitochondriate Pathogens

Like the SUF pathway, the mitochondrial ISC pathway was also inherited from an ancestral endosymbiont. The ISC pathway supplies FeS clusters to mitochondrial proteins, including components of the electron transport chain. In addition, maturation of cytosolic and nuclear FeS proteins is also dependent on the ISC pathway. It is thought that the ISC machinery exports a sulfur-containing moiety (the composition of which is still unknown) from the mitochondrial matrix to the cytosol, where it is further elaborated by the cytosolic CIA pathway [2]. Intriguingly, amitochondriate protists have retained the ability to synthesize FeS clusters in relict organelles, which include the hydrogenosome of *Trichomonas vaginalis* [10], the mitosomes of *Giardia intestinalis* [11] and *Entamoeba histolytica* [12], [13], and the ribosome-studded remnant mitochondrion of *Cryptosporidium parvum* [14]. Even among microsporidian pathogens, such as *Encephalitozoon cuniculi*, the mitosomes have been shown to contain key enzymes of FeS cluster synthesis [15]. These mitochondrion-like organelles (MLOs) are thought to share a common ancestor with mitochondria [16], and the amitochondriate state seems to be the result of reductive evolution rather than a primitive condition. MLOs are biochemically and structurally simplified compared to mitochondria, and generally do not contain an organellar genome. The unusual organelle of *Blastocystis* is an exception, however; it contains both cristae and its own genome, and energy metabolism that suggests it is an evolutionary intermediate between mitochondria and hydrogenosomes [17]. FeS cluster biogenesis seems to be the common thread linking mitochondria and MLOs, and is likely the selective force driving the retention of these organelles.

## FeS Cluster-Dependent Metabolism in Eukaryotic Pathogens

Why is there such absolute dependence on a compartmentalized ISC system? It has been suggested that the need to retain an organellar ISC pathway may reflect a reliance on a membrane potential for maturation of FeS cluster proteins [18]. But what are the essential downstream targets of the pathway? There does not seem to be any essential mitochondrial FeS protein that is conserved across all lineages, so the answer must lie outside of the organelle. In classical mitochondria, there are FeS proteins within the organelle that rely on the biogenesis machinery, including subunits of complexes I, II, and III of the electron transport chain and citric acid cycle enzymes such as aconitase. Trypanosomes depend on oxidative phosphorylation for ATP production, albeit only in certain stages of their life cycle [19], and the electron transport chain of *P. falciparum* is essential for the regeneration of ubiquinone for pyrimidine biosynthesis [20]. The trichomonad hydrogenosome also requires FeS proteins for energy metabolism. The FeS proteins ferredoxin and pyruvate∶ferredoxin oxidoreductase are involved in the fermentative metabolism of pyruvate and the formation of molecular hydrogen, which leads to the production of ATP through substrate-level phosphorylation [10]. In an interesting variation, *E. histolytica* has replaced its original mitochondrial ISC system with a bacterial NIF (NItrogen Fixation) pathway acquired by lateral gene transfer [12], [13]. In addition to its role in the cytosol, the NIF system has been adapted to function in mitosomes, possibly for the maturation of the FeS protein rubrerythrin (Rbr), a protein also present in hydrogenosomes [21] that is required for peroxide detoxification in these organelles [22]. However, there is no evidence for ATP production in the mitosomes of *G. lamblia*, *E. hisolytica*, *C. parvum*, or the microsporidia, and in some cases there are no known FeS-dependent proteins in these organelles. Thus, it is unlikely that FeS-dependent metabolism within mitochondria and MLOs accounts for the retention of FeS cluster biogenesis in these organelles. Instead, organellar FeS cluster synthesis is probably required for the maturation of FeS proteins in the cytosol and nucleus.

## The CIA Pathway Performs Critical Functions in the Cytosol and Nucleus

Perhaps the most extreme example of reductive evolution of the mitochondrion is found in the apicomplexan *C. parvum*. This organism is unusual among the apicomplexans in that it has completely dispensed with an apicoplast [23] and contains a relict mitochondrion that is structurally distinct from both mitosomes and hydrogenosomes [24]. This double-membrane-bound organelle is enveloped by the rough endoplasmic reticulum, and the only metabolic function ascribed to it thus far is the biosynthesis of FeS clusters [14], suggesting that the sole reason for retention of this organelle is the export of FeS clusters for use in the cytosol and nucleus. Indeed, components of the FeS export machinery as well as the cytosolic CIA pathway have been identified in the *C. parvum* genome [25]. One likely essential role for the ISC/CIA pathway in *C. parvum* is maturation of the RNase L inhibitor 1 (Rli1) protein, an essential cytosolic FeS protein common to all eukaryotes that functions in ribosome biogenesis and translation initiation [26], [27] and termination [28]. The connection between FeS cluster biogenesis in the relict mitochondrion and ribosome assembly and function would also explain the close association of the organelle with the rough endoplasmic reticulum.

There are two other nuclear FeS proteins that are essential in yeast and conserved across eukaryotes: Rad3, a helicase involved in nucleotide excision repair [29], and Pri2, which functions in RNA primer synthesis for DNA replication [30]. These three FeS proteins, and perhaps others not yet identified, seem to be the most ubiquitous feature of FeS cluster metabolism, and the essential functions they perform in the cytosol and nucleus may provide the driving force for retention of both the mitochondrial FeS synthesis pathway and the cytosolic CIA machinery in all eukaryotic pathogens. The universal requirement for FeS clusters highlights the reliance of these organisms on the acquisition and homeostasis of iron.
